# High-Performance Reconfigurable Pipeline Implementation for FPGA-Based SmartNIC

**DOI:** 10.3390/mi15040449

**Published:** 2024-03-27

**Authors:** Xiaoyong Song, Rui Lu, Zhichuan Guo

**Affiliations:** 1 National Network New Media Engineering Research Center, Institute of Acoustics, Chinese Academy of Sciences, No. 21, North Fourth Ring Road, Haidian District, Beijing 100190, China; songxy@dsp.ac.cn (X.S.); lur@dsp.ac.cn (R.L.); 2 School of Electronic, Electrical and Communication Engineering, University of Chinese Academy of Sciences, No. 19(A), Yuquan Road, Shijingshan District, Beijing 100049, China; 3 Suzhou Haiwang Network Technologies Co., Ltd., Suzhou 215163, China

**Keywords:** field programmable gate arrays (FPGA), pipeline, switch, SmartNIC, reconfigurable match-action table

## Abstract

As the key module of programmable switches or the SmartNIC card, the packet processing pipeline undertakes the task of packet forwarding and processing. However, the current pipeline for the FPGA-based SmartNIC is inflexible, and the related reconfigurable commercial device designs are closed-source. To solve this problem, this paper proposes a high-performance reconfigurable pipeline design, which has fully reconfigurable match-action units, supporting various network functions by its flexible reconfiguration. The fields of the match key and the size of the match table can be reconfigured without recompiling the HDL code or modifying the hardware. The processing rules and action instructions for the pipeline can be dynamically installed by the configuration module at runtime. We implement our design on the Xilinx Alveo U200 board with a Virtex UltraScale+ XCU200-2FSGD2104E FPGA and show that the designed pipeline supports fast reconfiguration to implement new network functions and that the throughput of the designed pipeline reaches 100 Gbps with low latency.

## 1. Introduction

As a classic architecture in computer hardware, the pipeline structure was originally developed for the execution of instructions [1,2]. The design of a pipeline is of great significance in improving the throughput of the processor and the efficiency of instruction execution, and it has been widely adopted in various computer designs [3]. An alternative model to the pipeline structure is the run-to-completion (RTC) model, where each task is completed before moving on to the next [4]. In contrast, the pipeline structure divides the entire processing task into multiple stages and executes independent subtasks in parallel within each stage, following a specific order.

In contemporary times, the pipeline structure is commonly employed in network devices, including SmartNICs, to enhance processing efficiency and throughput. The entire packet processing process involves parsing and organizing the data processing flow in a directed acyclic graph, following a particular order, which is then mapped into the processing pipeline [5]. Each stage of the pipeline is responsible for executing specific independent tasks, such as packet parsing, routing matching, rule matching, checksum calculation, and packet encapsulation.

With the improvement in network speeds and the enrichment of network scenarios, traditional networks face challenges in terms of flexibility and performance. SDN (software-defined networking) networks separate the data plane from the control plane, enabling greater flexibility [6]. However, the performance of current SDN software switches is limited, and the software switches consume a significant amount of the CPU resources of host servers. In recent years, offloading and accelerating network functions on SmartNIC have emerged as a popular approach [7].

In addition to the functions of the traditional network interface card (NIC), a SmartNIC card has a wide range of applications and significant importance in the acceleration of network function offloading. An FPGA-based SmartNIC is an important kind of SmartNIC. The offloading of network functions from the software switch to the FPGA-based SmartNIC can not only save server CPU resources but also realize faster processing or forwarding of packets. After receiving a data packet, the SmartNIC performs various operations on the packets as designed and forwards the packets after processing. In software-defined networking (SDN), the data plane is offloaded to hardware, providing high-throughput and low-latency packet processing and forwarding capabilities to optimize network performance. The packet processing unit in the data plane also often adopts a pipeline design to improve throughput and system frequency.

Even though FPGA has advantages in programmability, the hardware design and development on FPAG is often time-consuming and laborious. The inflexible data plane pipeline for an FPGA SmartNIC has problems especially in limited and fixed operation functions and efficiency of development and testing. For the design of large-scale FPGA hardware systems, the compilation of hardware code takes several hours. In the deployment of new functions or verification and testing of some new protocols, it is necessary to remodify the hardware code or recompile and debug, which is inefficient. The pipeline of the data plane is mainly organized by several match-action units (MAUs) with match- action tables (MATs). In most current FPGA-based designs, the MAT is fixed in width, depth, and type, and the actions performed in each MAU are also fixed. However, with emerging of network protocols and network scenarios, the flexibility and reconfigurability of a pipeline are increasingly required. For example, for packet forwarding processing applications in SDN networks, the main difference among different functions often lies in the forwarding match tables and the actions performed in the pipeline. If the requirement changes, remodifying the hardware code and recompiling the FPGA take quite a long time. If the pipeline in the SmartNIC data plane is reconfigurable, it only needs to quickly update the configuration register to modify the function of the packet processing pipeline and reconfigure the match-action units and other modules, which only takes a few seconds or milliseconds.

To solve these problems, this paper proposes a reconfigurable pipeline for an FPGA-based SmartNIC. The proposed pipeline can be reconfigured and reconstructed through the control plane without redesigning and developing the hardware code. Different from the way of communication in [8], our FPGA SmartNIC communicates with the control plane of the host server with PCIe. The user does not need to modify the hardware code or reburn a new bitstream file to the FPGA; rather, the user need only update the configuration registers to complete a new configuration. The match-action units and the match-action table for the designed pipeline can be reconfigured as user-defined, and the action instructions can be reconfigured to realize different network functions. The main contributions of this work are as follows:

- A complete reconfigurable pipeline scheme based on FPGA is proposed, and the proposed pipeline design can achieve 100 Gbps throughput with low latency.
- The MAU for the proposed pipeline has full reconfigurability in its match field, table size, table type, and action instructions. As we know, previous FPGA-based schemes may have some but not all of these features.

## 2. Related Work

Hardware-based SmartNICs or programmable switches not only require high performance, but also need to exhibit flexibility to accommodate various scenarios. The packet processing pipeline plays a critical role in the data plane, particularly in terms of its reconfigurability to handle different types of packets.

In the protocol-independent switch architecture (PISA) [9] and the architecture of P4 [10], the “Match + Action” abstraction is employed to handle packet processing, with the processing pipeline organized using multiple match-action tables. The single match-action table (SMT) structure allows for pipeline packet processing within a single match-action table, but it has limited scalability. This limitation arises from the exponential increase in the number of rules within the match table when multiple fields are used as the match field simultaneously [11,12].

The multiple match-action table (MMT) structure offers greater flexibility and effectively reduces the entry space requirement by employing multiple processing stages. In the case of the MMT structure, there are implementations like AccelSDP [13] or the design in [14] that utilize a multi-stage pipeline with several match-action tables. However, these implementations lack support for reconfiguring the match table and the pipeline itself. AccelSDP [13] implements two match stages in its pipeline and only allows a small number of predetermined packet operations within a specific stage. This restricted configurability limits its flexibility. The pipeline described in [14] supports a greater number of stages, and the action performed within each stage can be configured with a controller. This provides more flexibility compared to AccelSDP. However, the drawback is that the size of the match table cannot be modified. This can result in wasted match resources and limits the overall flexibility of the pipeline.

The reconfigurable match-action table (RMT) [12] structure was proposed to address the disadvantages of the SMT and MMT structures. It has been widely adopted by designs such as FlexNIC [15,16] and PANIC [17]. The RMT pipeline consists of multiple stages, with reconfigurable match-action tables in each stage. The “reconfigurable” aspect means that the size, type, and number of match-action tables can be reconstructed as needed. This flexibility allows better customization and adaptation related to different requirements. The action engine in the RMT architecture includes a set of ALUs (arithmetic logic units) that support various action instructions, providing powerful processing performance and flexible computing capabilities. It uses a VLIW (very long instruction words) to process multiple PHV (packet header vector) containers simultaneously in each stage. However, most of the RMT pipeline design is ASIC-based and unclosed, such as Intel Barefoot Tofino [18] and FM6000 [19]. Menshen [20] implemented RMT on FPGA, and it has the reconfigurability of search keys and action instructions, etc. However, its match table is still fixed, which limits the flexibility of the pipeline. Drawerpipe [21] has good flexibility, and it can quickly reconstruct a pipeline by recombining the developed hardware modules of the SmartNIC pipeline, but its performance and efficiency are low.

Moreover, in the design of a reconfigurable pipeline, the crossbar is commonly used in many modules to achieve higher flexibility and reconfigurability. The crossbar provides a high degree of connectivity, allowing more versatile routing and data flow between different components in the pipeline. The choice of the specific connectivity structure or technique depends on various factors such as the target application, performance requirements, power constraints, and design complexity. Different designs may opt for alternative solutions based on their specific needs and trade-offs. By limiting the unit and range of selection fields, resource consumption can be effectively reduced while ensuring rationality [22]. In the work of [23], an efficient header analysis and field extraction architecture is proposed for FPGA-based network applications. In the design of dRMT [24], to reduce the overhead of the input crossbar in the action engine, only 32 ALU parallel operations are present in each pipeline stage, and the corresponding fields are written back to the PHV through the output crossbar. If processing is required for more than 32 fields in the PHV, it is done several times, which saves a significant portion of the crossbar overhead.

## 3. System Design

### 3.1. Hardware Architecture

The processing pipeline consists of multiple stages with match-action units, and the key module of the reconfigurable pipeline is the reconfigurable match-action unit. In this section, we describe the design of our pipeline and the MAU.

**Figure 1:**
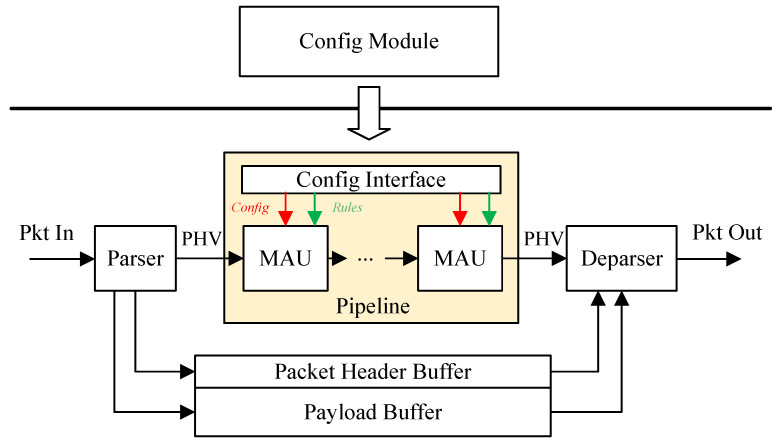
Architecture of the system.

As shown in Figure 1, in the packet processing system, the PHV of the packet is generated by parser firstly, and then it enters the pipeline for processing. After the pipeline processing, the processed PHV enters into deparser and is combined with a payload to generate the processed packet. The focus of this paper is mainly in the yellow field of Figure 1, which includes a reconfigurable pipeline, the reconfigurable MAU, and the config interface to transform configuration information from the control plane to the hardware module in FPGA.

**Figure 2:**
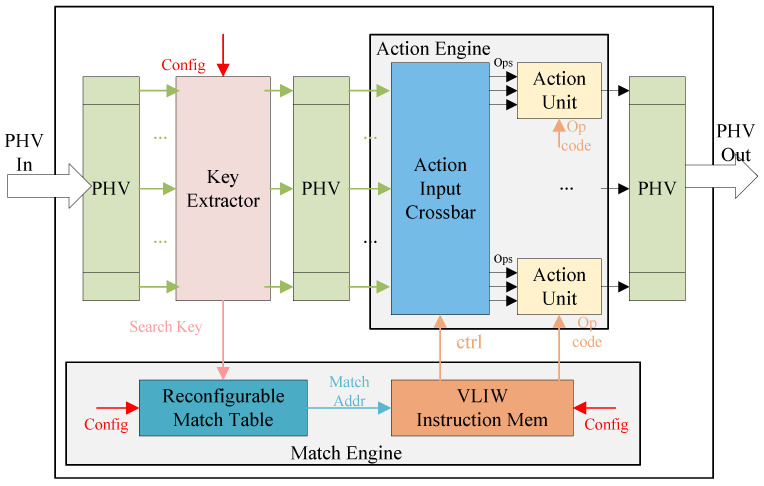
Architecture of the MAU.

The processing pipeline consists of multiple MAUs. As shown in Figure 2, the match-action unit in each stage can be reconfigured, including the field and the length of the search key, the number and size of the match table, and the action instructions to be executed. Each MAU has a reconfigurable key extractor, reconfigurable match engine, and reconfigurable action engine. The key extractor realizes different fields or different lengths of the search key; then, the search key obtains match results with the match engine, and the action engine performs operations on the PHV according to the match results and action instructions.

### 3.2. Packet Header Vector

The PHV transverses all modules in the processing system such as the packet parser, pipeline, and deparser. It is made of the packet header information and the metadata such as intermediate information and temporary values in the processing process, and it is organized with several containers. In order to reduce the resource consumption of FPGA, the PHV in the designed system only contains the information that needs to be processed rather than all the packet header information. After the parsing of a packet, the original packet header is not removed. The whole packet is stored in the packet buffer, and the deparser updates or modifies the packet header based on the processed PHV.

**Figure 3:**
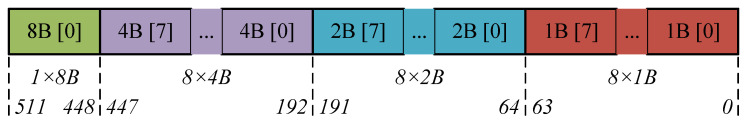
The architecture of the PHV.

As Figure 3 shows, in our design, the PHV has an overall width of 512 bits, which is divided into four types of containers, totaling 25 containers. These containers consist of eight 8-bit containers, eight 16-bit containers, eight 32-bit containers, and one 64-bit container. During the process of parsing, the packet header fields are placed into the defined container based on the configuration information. The first 24 containers are primarily used to store packet header fields. Moreover, the 64-bit container is specially designated to store specific information, such as the flag of whether the packet should be forwarded, indicated by the [0] bit. Other fields in the 64-bit container are configured or reserved as required.

### 3.3. Key Extractor

The key extractor for each match-action unit can be configured independently by the control plane. According to the configuration information, the specified fields in the PHV are selected and combined as the search key. The search key generated by each key extractor can be combined with different fields and have different lengths. The key extractor in our design can generate up to two search keys in each stage, with one for the exact match table and the other for the TCAM table. Each search key is composed of up to four container fields in the PHV, that is, the maximum width of a single search key is 128 bits.

**Figure 4:**
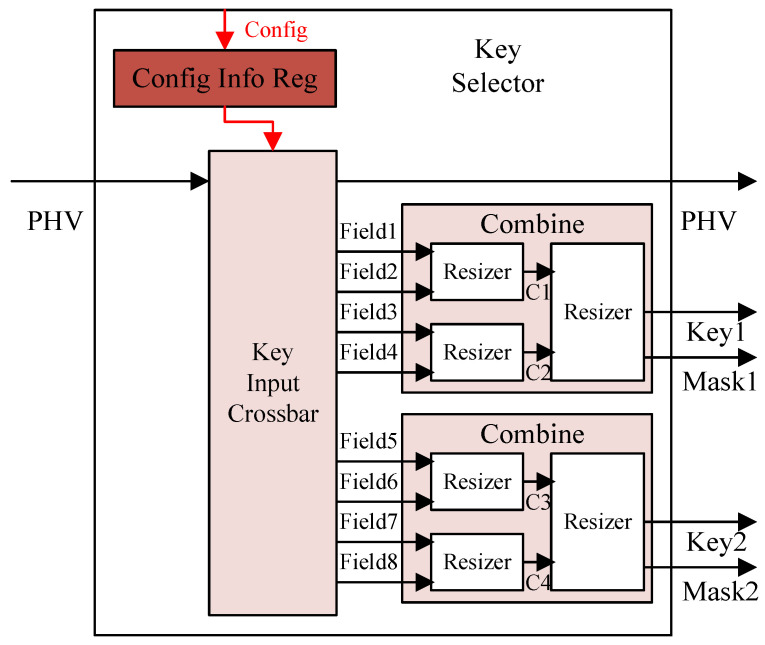
Configurable key extractor.

As Figure 4 shows, the key extractor is mainly composed of the key input crossbar and the combine module, which are used to select the corresponding container fields from the PHV and combine them as the needed key. The config module configures the key information for the exact match and TCAM match in each match-action unit before it works. The configuration information includes the type and the index of the container to be selected, the starting position, and the offset length of the extract field in a container. After the key input crossbar extracts the PHV fields based on configuration information, it selects the fields from four specified containers for each search key. The combine module then combines the valid parts of the four fields corresponding to the exact match and the TCAM match, respectively, to generate the respective search keys and sends them to the match engine for query.

In the combine module, a 2-level method is used to combine and shape fields in pairs through multiple resizers. The resizer selects the valid parts from the two input data, outputs the merged key, and generates a mask based on the valid key length. The mask is used to represent the valid field of the search key to support the variable length key.

### 3.4. Match Engine

**Figure 5:**
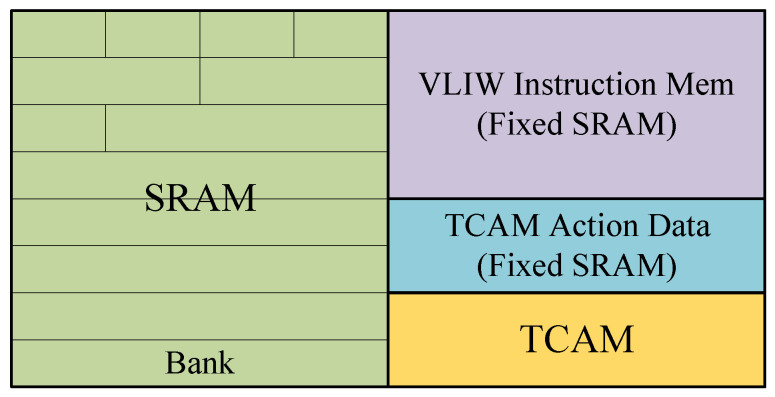
Memory for a single stage.

The match engine responds to the query of the search key to obtain the action data and instructions used by the action engine. It is mainly composed of match tables and memory storage. As shown in Figure 5, there are TCAM resources and SRAM resources in each pipeline stage MAU for the design of the match table and the storage of other information like action data and instructions. At most, one TCAM logical match table and one exact match table based on hash can be implemented in each stage. TCAM resources within a single stage can only be configured as a single TCAM logical table or configured as an exact match table in special cases. The SRAM resources include reconfigurable SRAM resources and dedicated storage resources. Reconfigurable SRAM resources are used to implement the user-defined exact match table and its action data table, and dedicated storage resources are used to store the action data of TCAM and instructions of TCAM and EM. It should be noted that because the number of action instructions is relatively small, specific resources are allocated for storage. Similarly, because the TCAM depth implemented in our system is relatively small, dedicated storage resources are directly allocated to store TCAM table action data. In the case of a large TCAM depth, memory storage can be allocated from configurable SRAM resources for its action data. In addition, since the exact match table and the TCAM logical table in each pipeline stage can work parallelly, it is necessary to ensure that their instruction actions are completely independent.

**Figure 6:**
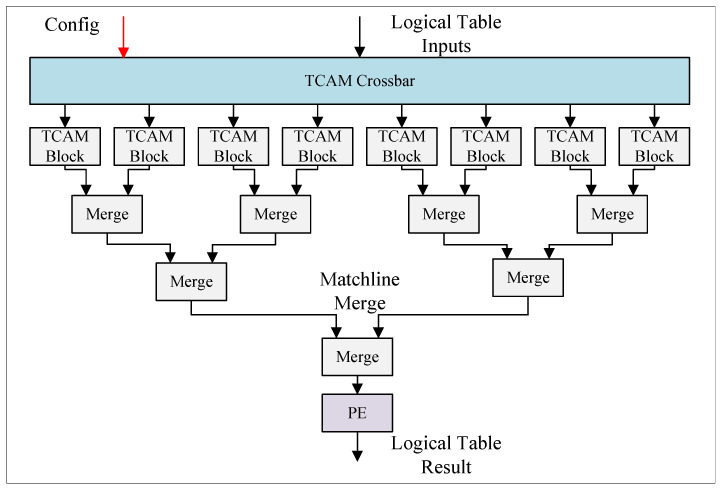
Reconfigurable TCAM.

As shown in Figure 6, the reconfigurable TCAM match table in each MAU consists of a TCAM crossbar, TCAM blocks, match-line merge blocks, and a priority encoder. After the configuration of the TCAM crossbar, it is responsible for converting the logical table input signals into each TCAM block. In the query process of TCAM, the match lines of all TCAM blocks are combined, and the final match address of the logic TCAM is obtained from the priority encoder. The reconfigurable TCAM resources in each stage include eight 32b × 32 TCAM blocks. Corresponding to the maximum length that can be extracted by the key extractor, the maximum width of the TCAM logical table can be configured to 128 bits. The logical table can be configured as 32b × 256, 64b × 128, 96b × 64, or 128b × 64 as required. According to the TCAM logical table width and depth configuration, TCAM blocks are cascaded, combined, and organized into the required size. The implementation of the TCAM block is independent of the reconfigurable design, and TCAM blocks here are flip-flop based [25].

A reconfigurable SRAM resource is used for the implementation of the exact match table and the storage of the action data of the EM table. The reconfigurable SRAM resource in a single MAU can be configured as two logical tables, an EM table, and its action data memory. The reconfigurable SRAM resource consists of 32 32b × 1K BRAM and is organized into eight 128b × 1K banks; the bank is the minimum unit for allocation or usage. The maximum width supported by a single logical table is 128 bits. Each bank can be configured in four modes: 32b × 4K, 64b × 2K, 96b × 1K, or 128b × 1K, which defaults to a 128 × 1K configuration.

According to the depth and width of logical tables, it allocates memory banks to each table. The SRAM crossbar is used for signal mapping conversion to realize the interconnection between logical and physical tables. The width configuration of the SRAM logical table is done within the banks. Each bank has a read or write width of 128 bits. However, each address space contains four 32b slots, and partial data can be written in a single address space. That is, a single write of 32b, 64b, 96b, or 128b width data is supported. The depth of the bank is cascaded to realize the wanted depth of the SRAM logical table.

After configuration, each logical table has its bank set as [Bank_Sid, Bank_Eid]. When the logical table is written or read, each interface signal is mapped and transformed as Table 1. The read and write address contains three levels of address fields. The first level is the index of the bank (Bank_id), which is used to determine the bank block to be accessed. The length of Bank_id here is 3 bits, corresponding to eight bank blocks. The second level is the address space index (SRAM_id) in the bank block, which is used to index the 128-bit address space in a bank. The length of SRAM_id is 12 bits, corresponding to the depth of 4K. The third level is the slot index (Slot_id) in a single address space of the bank, which is used to locate the slots in a single address space. The width of Slot_id is 0 to 2 bits and is determined by the width configuration of the logical table and the width of the slot. The start bank of a logical table (Bank_Sid) corresponds to the base address of the storage space in the logical table. When it accesses a logical table, it needs to determine which bank to access (Bank_id) and obtain the bank address (SRAM_id) according to the base address and the original address of logical table (lw_addr). The write data (Bank_data) and the mask (Bank_mask) are generated by the original data (lw_data) and the width of the logical table.

In addition to the exact match table and the TCAM match table, there are action data memory and instruction memory. In this design, each match table (EM or TCAM) is equipped with an action instruction memory and an action data memory. The depth of each instruction storage unit is 32, and the width is the same as VLIW. After the EM and TCAM match table are queried, the action data are obtained from the action data store according to their match addresses. In the designed system, the lowest 5 bits of the action data represent the instruction address, which is used to obtain the VLIW instruction from its memory. The remaining fields can store the operands and other information outside the instruction as needed. The VLIW instructions of EM and the TCAM are merged to obtain all the VLIW instructions for a stage. Both VLIWs indicate that some fields in the PHV are operated, but the actions performed by two tables in the same pipeline stage should be completely independent. The invalid field in the instruction is set to “0”. Similarly, the action data of EM and TCAM are merged and entered into the action engine together with the PHV and VLIW instructions, and the corresponding actions are performed on the containers in the PHV.

### 3.5. Action Engine

As shown in Figure 2, the action engine consists of an action input crossbar and a set of arithmetic logic units (ALUs). The action input crossbar is used to extract the operands and the opcode of ALUs from PHV, action data, and VLIW. There are 25 ALUs in each action engine, which correspond to the 25 containers in the designed PHV. Therefore, it is possible to operate on all PHV containers in a single stage simultaneously. To reduce the overhead of the crossbar, the input crossbar is divided into the PHV crossbar and the action data crossbar. The PHV crossbar primarily extracts fields from the PHV containers and immediate values from the instructions as operands. The action data crossbar primarily extracts operands from the action data.

The action instructions are organized as VLIW and stored in specific storage within the match engine. Each VLIW consists of multiple independent action instructions. Based on the operation code and operands in the action instructions, the ALUs perform the corresponding arithmetic operations and output the results, which are then written back to the corresponding containers in the PHV. Just like the container type of PHV, the ALUs are divided into 8-bit, 16-bit, and 32-bit ALUs, and a specific ALU handles the remaining fields in the PHV.

**Figure 7:**
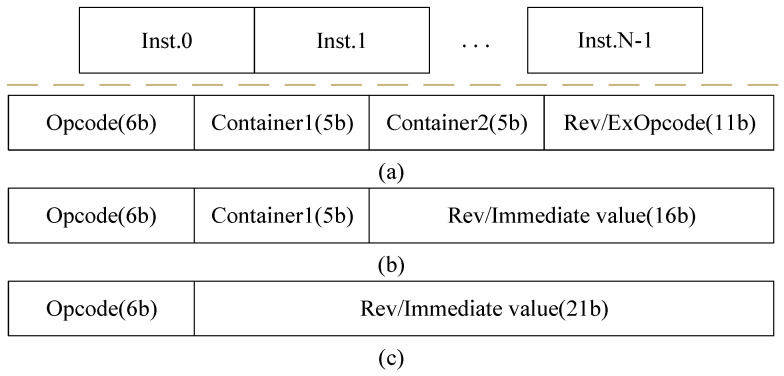
The design of VLIW and instructions. (**a**) Instruction with 2 operands from PHV container. (**b**) Instruction with 1 operand from PHV container. (**c**) Instruction with 0 operand from PHV container.

The instructions supported are mainly divided into register-based instructions and immediate-based instructions. Each instruction has a length of 27 bits, including a 6-bit operation code. There is no need for a destination address in our instructions since the ALUs and PHV are hard-wired in our action engine, meaning that the destination address for the ALU output is determined. In Figure 7a, the instruction contains two container fields, indicating that both operands of this instruction are from PHV containers. Each field indicates that the index of the container is 5 bits. The upper 2 bits represent the container type, with 2’b00 representing an 8-bit container, 2’b01 representing a 16-bit container, 2’b10 representing a 32-bit container, and 2’b11 representing a 64-bit container. The remaining 3 bits represent the container position, corresponding to a maximum of 8 containers from 0 to 7. In Figure 7b, the instruction contains one container field and one immediate field. The immediate field can support up to 16 bits. Operand2 may come from the immediate value in the instruction or the action data. In Figure 7c, the instruction only contains an immediate field. Operand1 of this instruction may come from the immediate value in the instruction or the action data, and it can be a combination of both.

As shown in Table 2, the supported action instructions in the current design mainly include logical and arithmetic operations on registers or immediate values as well as specific processing of data packets such as TTL decrease by 1, setting port numbers, discarding or forwarding data packets, and so on.

All operations in the action engine are unidirectional pipelined. The action engine does operations for PHV containers and generates a new PHV, which is then passed to the next stage or MAU in the pipeline for further processing. Multiple stages are connected in the processing pipeline, and the deparser combines the processed PHV with the packet payload to a new packet.

### 3.6. Config Module

In the designed system, the pipeline in the FPGA hardware data plane is reconfigurable. Its reconfigurability is reflected in two aspects. Firstly, the match-action units in the pipeline are fully reconfigurable, including the fields and lengths of search keys, the size and type of the match tables, etc. Secondly, the configurability is exhibited in the data packet processing rules, including the matching rules of the match tables, the action data, and the processing of action instructions. The former is statically configured, meaning that the data plane needs to be reset if the pipeline is reconstructed. The latter is dynamically configured, meaning that processing rules and action instructions can be installed in runtime.

Communication between the configuration module and hardware is via PCIe. The generation of configuration information or processing rules is not the focus of this work, so we do not discuss it here. Here, we configure this by configuring registers and assume that this information is already generated. After receiving the information, the configuration interface module in the hardware system converts it into the required signals and sends them to the match-action units in each pipeline stage. After each configuration is completed, the configuration information memory in each module stores the information and uses it until the next reset or update.

## 4. Implementation and Evaluation

### 4.1. Implementation

A designed reconfigurable pipeline together with a packet buffer, packet parser, and deparser form the packet processing system. Each pipeline stage has reconfigurable TCAM resources, including 8 32b × 32 TCAM blocks; reconfigurable SRAM resources include 32 32b × 4K SRAM blocks. The packet buffer is 1MB.

The system is integrated into Corundum [26] for implementation and testing. We developed the system and pipeline with Verilog and implemented it on the Xilinx Alveo U200 [27] board. There is a Virtex UltraScale+ XCU200-2FSGD2104E FPGA (16 nm) on the AU200 board, and it has 1,182,240 LUTs, 591,840 LUTRAM, 2,364,480 FF, 2160 BRAM36K, and 960 URAM on the FPGA. The data width of the AXIS interface is 512 bits, and the system frequency is 250 MHz. The configuration module and devmem file work on the server. The AXI-Lite interface is used to transmit control signals on the FPGA, configure the pipeline, and install processing rules.

The AU200 FPGA is deployed on the Dell R730 commercial server and communicates with the FPGA through PCIe. A QSFP28 Ethernet transceiver in the U200 and a 100 Gbps optical port of the IXIA XGS12 network tester are connected via fiber optics. After the control plane sends configuration information, the IXIA tester sends data packets to the FPGA for processing. The processed data packets are output from the FPGA and sent back to the IXIA tester.

### 4.2. Use Cases of the L2/L3 Switch

When the pipeline is not configured or reset, the default pipeline only forwards packets like the Corundum framework without any extra processing. In addition, in this section, a 4-stage pipeline processing system is used to verify its reconfigurability through configurations of the pipeline to achieve the function of the L2/L3 switch with different match-table sizes, processing rules, etc.

In the network, L2/L3 switches in packet forwarding and routing, the match fields include an Ethernet type, a destination IP address, a source MAC address, and a destination MAC address. A combination of these match fields is typically used to match the routing table, and based on the match results, routing decisions are made about from which interface to forward packets.

We configure the pipeline by the size and the order of the match table. The MAU in the 1st stage is configured as an exact match table with the source MAC address as its match field, and the logical table size is 48b × 4K. The key extractor extracts a 16-bit container and a 32-bit container from the PHV to generate its search key. The MAU in the 2nd stage is configured as an exact match table that is the same as the former one, whose match field is the destination MAC address. The MAU of the 3rd stage is configured as a 32b × 256 TCAM table to match the IPv4 destination address. The search key is extracted from a 32-bit container of the PHV. The MAU of the 4th stage is configured as a 128b × 64 TCAM table with the IPv6 destination address as the match field. The key extractor extracts the corresponding four 32-bit container fields from the PHV and combines them to form the query key.

**Figure 8:**
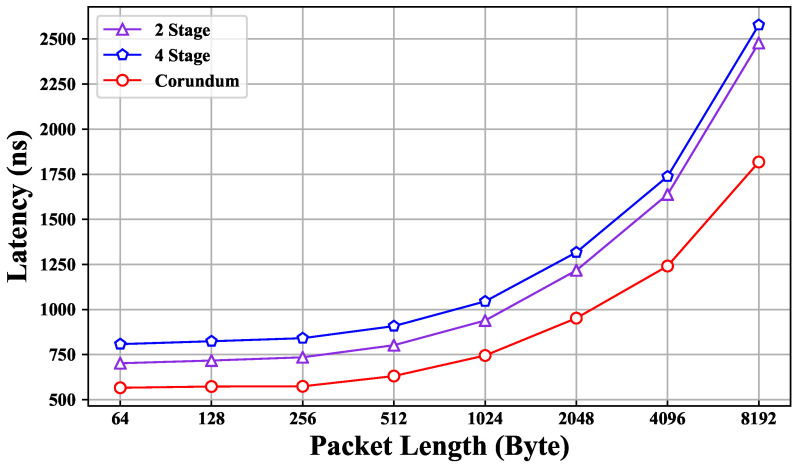
Latency test.

A 100 Gbps optical port on the IXIA instrument connects to the QSFP0 port on the Alveo U200 via an optical fiber, and the FPGA forwards the packet directly from the original port back to the IXIA after receiving and processing it. The throughput of the system with a single port is 100 Gbps. As shown in Figure 8, overall, the packet processing system has low latency. For packets under 4096 bytes, the latency is less than 2 μs. Compared to the Corundum prototype, the proposed application increases the processing delay by about 1 μs and is correlated with the stage number of the pipeline. The processing latency increases with the number of pipeline stages, and the processing of each stage is eleven clock cycles, including one clock cycle for the key extraction, seven clock cycles for the match engine, two clock cycles for the action engine, and one clock cycle delay for the output. That is, in this implementation, each additional stage theoretically increases the processing delay by 44 ns.

### 4.3. Use Cases of the Packet Filter

#### 4.3.1. ACL

The 5-tuple of a packet is usually used to describe its characteristics and is used as the match field of ACL (Access Control List) for traffic access control. The five fields include the following: the source IP address, destination IP address, transport layer protocol, source port, and destination port. The ACL can match and control the traffic direction based on the values of these fields, or it can deny access.

The 5-tuple of IPv4 packets is used as the matching rule of the ACL, and its total width is 104 bits. In the process of configuration, the packet parser places the IPv4 source address and destination address into a 32-bit container, respectively. The transport layer protocol field is placed into an 8-bit container, and the source port and destination port are combined and placed into a 32-bit container. Bit [0] of the 64-bit reserved field in PHV is set as the Deny Flag, indicating whether a packet is allowed to pass or deny. Because there are IP fields, wildcard matching needs to be performed using the TCAM match table. We configured a 104b × 128 TCAM in each stage, and the key extractor is configured to extract the specified 4 containers (three 32-bit containers and one 8-bit container) and combine them to obtain the search key. Four TCAM tables in the four pipeline stages are cascaded to a 104b × 512 TCAM table. The ACL table with rules actually is a blacklist. If a match succeeds, the Deny Flag field in the PHV is marked as 1, indicating that the packet is denied, and it would be discarded in the outport.

As shown in Table 3, we use the 5-tuple of the packet as the metric for packet forwarding. The IXIA tester generates four types of packets with different packet headers and sends them into the packet processing pipeline of the FPGA SmartNIC. All packets can pass through the packet processing pipeline before ACL rules are installed. After the installation of ACL rules, the successfully matched packets are discarded and cannot be forwarded. More details of the experiment are presented in Appendix A.

#### 4.3.2. Multi-Stage Packet Filter

It also could map the user-defined table into the pipeline with other methods and configure the pipeline as a multi-stage packet filter to implement the ACL. It could divide the ACL rule as four subfields and configure four match tables in the 4-stage pipeline. Moreover, it also could divide the match field into two subfields. For example, the source IP address and destination IP address are combined to perform ternary matching, and the TCAM in the MAU is configured with a width of 64b; the protocol type, source port, and destination port are combined to perform exact matching, and the exact table in the MAU is configured with a width of 40b. The reserved field in PHV could be defined to indicate whether a packet should be sent or discarded.

### 4.4. Resource Utilization

The designed reconfigurable pipeline and processing system are integrated into the Corundum. Table 4 shows the system resource consumption with different pipeline stages. The framework of the Corundum consumes about 6.07% onboard LUT resources, and the packet parser, packet deparser, and packet buffer in our system consume approximately 1.43% LUT resources of Alveo U200. Beyond that, the extra resources are consumed primarily by the multi-stage pipeline. As Table 4 shows, the designed pipeline has good scalability for the number of stages. In the system integrated with the 8-stage pipeline, it occupies in total about 21.13% of LUT resources of the whole board, and other resource consumption is no more than 18%, which leaves enough resource and development space for other applications.

The resource utilization of a single MAU in the pipeline is shown in Table 5. Logical resources are mainly consumed by the TCAM blocks and various crossbars. The TCAM block here is implemented with flip-flops. In each MAU, there are eight 32b × 32 TCAM blocks, and eight 128b × 4K SRAM blocks to implement the match tables. It has an additional two 675b × 32 SRAM to store the VLIW of the TCAM table and EM table and a 128b × 256 SRAM to store the action data of the TCAM table.

### 4.5. Comprehensive Evaluation

As shown in Table 6, the proposed design has advantages in both performance and flexibility compared with other FPGA-based designs. Although the data plane of Drawerpipe has good flexibility and reconfigurability, its performance is very low and cannot meet the demand of a high-speed network. The work of AccelSDP [13] and M. Sha et al. [14] both have high throughput, but their pipelines have limited flexibility. In the AccelSDP pipeline, the function of each stage is fixed, and the size and type of the match-action table is unchanged. The pipeline of M. Sha et al. [14] has higher flexibility than AccelSDP, and the ALU could perform some preset actions according the configuration, but its flexibility is still limited and its match tables are solidified. Compared with these methods, the designs of our work and Menshen’s have more flexibility and more powerful packet processing.

Both our work and Menshen’s [20] perform actions to multiple containers parallelly in PHV with VILW, which is more powerful and flexible in the actions, and both of them have 100 Gbps throughput. However, our approach is more flexible especially in the match tables. Although the key extractor of Menshen could extract a search key with variable lengths, its match-action table is fixed in width and type. In each stage of Menshen’s pipeline, there is only a small fixed EM table implemented by CAM, while in our pipeline, each stage supports two independent match-action tables. There are a reconfigurable EM table and a reconfigurable TCAM table in each MAU, and the size of our tables is also larger than Menshen’s. We replaced Menshen’s CAM IP module with our CAM block and implemented both of them on the Xilinx AU200. A comparison of the single stage for the two methods is shown in Table 7. In contrast, our work has more advantages in terms of resource richness and flexibility of the match tables.

## 5. Conclusions

In this paper, we proposed a reconfigurable pipeline and successfully integrated and implemented it with Corundum on the Xilinx Alveo U200 FPGA. All key modules of the MAU in the pipeline could be reconfigured as defined, and the processing rules and action instructions could be installed dynamically. The proposed reconfigurable pipeline has high performance and flexibility and a 100 Gbps throughput for packet processing with low latency.

## Figures and Tables

**Table 1:** Mapping table of the SRAM signal.

Logical Table Width	slot_id	Access Bank_id	Access Bank_addr	Access Bank_data	Access Bank_mask
32b	2’b00	Bank_Sid+lw_addr[12:14]	lw_addr[11:2]	{96’h0,lw_data[31:0]}	16’h000F
32b	2’b01	Bank_Sid+lw_addr[12:14]	lw_addr[11:2]	{64’h0,lw_data[31:0],32’h0}	16’h00F0
32b	2’b10	Bank_Sid+lw_addr[12:14]	lw_addr[11:2]	{32’h0,lw_data[31:0],64’h0}	16’h0F00
32b	2’b11	Bank_Sid+lw_addr[12:14]	lw_addr[11:2]	{lw_data[31:0],96’h0}	16’hF000
64b	1’b0	Bank_Sid+lw_addr[11:13]	lw_addr[10:1]	{64’h0,lw_data[63:0]}	16’h00FF
64b	1’b1	Bank_Sid+lw_addr[11:13]	lw_addr[10:1]	{lw_data[63:0],64’h0}	16’hFF00
96b	-	Bank_Sid+lw_addr[10:12]	lw_addr[9:0]	{32’h0,lw_data[95:0]}	16’h0FFF
128b	-	Bank_Sid+lw_addr[10:12]	lw_addr[9:0]	lw_data[127:0]	16’hFFFF

**Table 2:** Supported operations in ALU.

Action	Description
add/sub	Two operands are both from PHV containers.
addi/subi	One operand is from a PHV container, and the other is
	an immediate value from the instruction or action data.
set field	Set a field with a specific value. The operand is from the instruction or action data or both.
or/xor	
sll/srl	Shift left or right. Two operands are both from PHV containers.
slli/srli	Shift left or right immediate. One operand is from a PHV container, and the other is
	an immediate value from the instruction or action data.
sub-1	TTL-1.
Outport	Set outport.
drop	Discard specific packets.

**Table 3:** ACL test.

Metrics (Five Tuple)		Test 1	Test 2	
		Before Installing ACL Rules	After Installing ACL Rules	
		Packet Forwarding	Match Success	Packet Forwarding
Packet Type	A	✔	✔	✘
	B	✔	✘	✔
	C	✔	✔	✘
	D	✔	✘	✔

A, B, C and D represent four different types of packets with different packet headers. ✔ means success, and ✘ means failed.

**Table 4:** Resource utilization for the whole system on the U200 FPGA.

System	LUT	LUTRAM	FF	BRAM	URAM
Corundum [26]	71,704 (6.07%)	12,877 (2.18%)	115,129 (4.87%)	141 (6.53%)	34 (3.54%)
2-stage Pipeline with Corundum	130,865 (11.07%)	15,221 (2.57%)	178,711 (7.56%)	160.5 (7.43%)	75 (7.81%)
4-stage Pipeline with Corundum	170,351 (14.41%)	17,447 (2.95%)	226,967 (9.60%)	164.5 (7.62%)	107 (11.15%)
6-stage Pipeline with Corundum	209,880 (17.75%)	19,673 (3.32%)	275,791 (11.66%)	168.5 (7.80%)	139 (14.48%)
8-stage Pipeline with Corundum	249,794 (21.13%)	21,899 (3.70%)	323,485 (13.68%)	172.5 (7.99%)	171 (17.81%)

**Table 5:** Resource utilization for a single MAU on the U200 FPGA.

Module	LUT	FF	BRAM	URAM
Match-Action Engine	23,676 (2.00%)	25,775 (1.09%)	2 (0.09%)	16 (1.67%)
Key Selector	4003 (0.34%)	274 (0.01%)	0	0
Match Engine	11,417 (0.97%)	21,561 (0.91%)	2 (0.09%)	16 (1.67%)
Action Engine	7486 (0.63%)	2880 (0.12%)	0	0
TCAM Crossbar	1050 (0.09%)	0	0	0
SRAM Crossbar	3052 (0.26%)	1553 (0.07%)	0	0
Action Input Crossbar	3050 (0.26%)	1830 (0.07%)	0	0
TCAM Pool	5632 (0.48%)	16,904 (0.71%)	0	0
SRAM Pool	0	1040 (0.04%)	0	16 (1.67%)

**Table 6:** Comparison of FPGA-based designs.

Work	Platform	LUT	BRAM	Freq	Throughput	Pipeline	Note about Action and
		/ALM	|URAM	(MHz)	(Gbps)	Flexibility	MAT in Pipeline
							Action in each stage is
AccelSDP [13]	Intel N3000	19.7%	9.2%|-	200	100	Low	fixed.
							MAT is fixed.
							Actions could be config-
M. Sha et al. [14]	Xilinx XCZU19EG	41%	35.72%|-	250	100	Mid	ured with SIMD.
							MAT is fixed.
							Actions could be config-
Menshen [20]	Xilinx AU250	13.65%	11.75%|-	250	100	Mid	ured with VLIW.
							MAT is fixed.
							Actions could be config-
Our work	Xilinx AU200	14.41%	7.62%|11.15%	250	100	High	ured with VLIW.
							MAT is reconfigurable.

The platform of the Intel N3000 has an Arria 10 (1150GT) FPGA (20 nm), and it has 1,150,000 logic elements, 427,200 ALMs, and 2713 M20K memory blocks. The platform of the Xilinx XCZU19EG FPGA (16 nm) has 522,720 LUTs, 984 BRAMs, and 128 URAMs. The platform of the Xilinx AU250 has a Virtex UltraScale+ XCU250-2LFIGD2104E FPGA (16 nm), and it has 1,728,000 LUTs, 2688 BRAMs, and 1280 URAMs. The platform of the Xilinx AU200 board has a Virtex UltraScale+ XCU200-2FSGD2104E FPGA (16 nm), and it has 1,182,240 LUTs, 2160 BRAMs, and 960 URAMs.

**Table 7:** Comparison the MAU in a single stage.

Work	LUT	FF	RAM D/B/U	Processing Latency	Memory Size	MAT
Menshen [20]	11,566	24,275	536/0/0	8 cycles	205b × 16 TCAM	A fixed TCAM-based EM table.
					625b × 16 SRAM	
Our work	23,676	25,775	1434/2/16	11 cycles	8 × (32b × 32) TCAM	A reconfigurable TCAM table.
					8 × (128b × 4K) SRAM	A reconfigurable EM and its action data memory.
					2 × (675b × 32) SRAM	Two fixed VLIW memory.
					128b × 256 SRAM	A fixed TCAM action data memory.

D means distributed RAM, and it means LUTRAM here. B means block RAM. U means ultra RAM.

**Figure A1:**
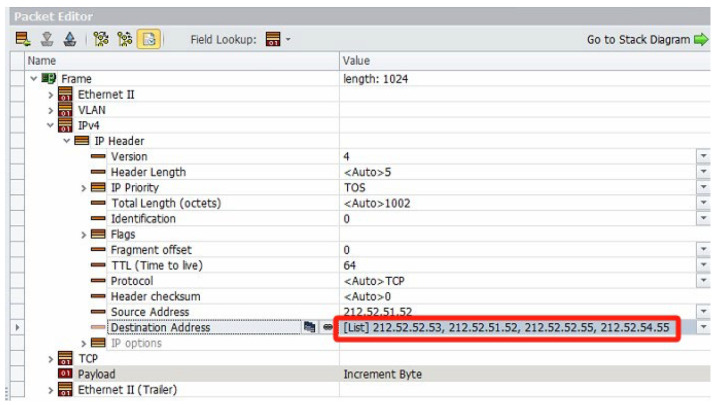
Packet configuration for testing with the IXIA tester.

**Figure A2:**
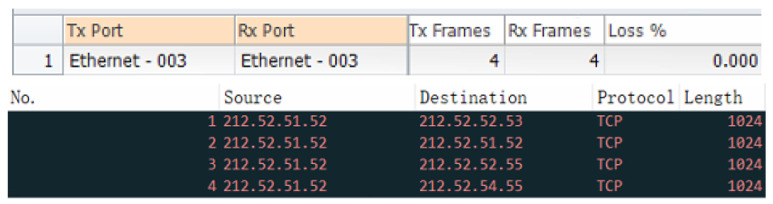
Test before installing ACL rules.

**Figure A3:**
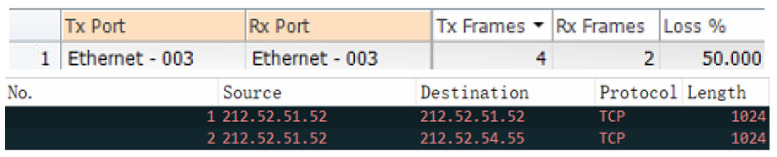
Test after installing ACL rules.

## Data Availability

The original contributions presented in the study are included in the article, further inquiries can be directed to the corresponding author.

## Appendix A

This appendix is used to further detail the ACL packet filtering experiment in Table 3.

After the pipeline configuration, we install the ACL rules and action instructions into the TCAM tables. Some rules are shown in Table A1, and if the match succeeds, the Deny Flag in the PHV would be modified to “1”. The IXIA tester generates data packets as shown in Figure A1. The destination IP address list of the data packets includes [32’hd434_3434, 32’hd434_3432, 32’hd434_3434, and 32’hd434_3434]. Four packets with different DIP are sent, and two packets are matched, and packets that match rules in the ACL match table are filtered out.

**Table A1:** Rules in the ACL table.

No.	Match Field <SIPv4,DIPv4,Protcol,SPort,DPort>
1	<32’hd434_3334/24, 32’hd434_3332/24,8’d17,16’h0001,16’h0002>
2	<32’hd434_3334/24, 32’hd434_3334/28,8’d 6,16’h0011,16’h0202>
3	<32’hd434_3334/32, 32’hd434_3334/32,8’d17,16’h0001,16’h0002>

As Figure A2 shows, before installing the ACL rules, packets with four different headers can be received without loss. After the rules listed in Table A1 are installed, the matched packets are discarded, and only two packets are forwarded out the pipeline in Figure A3.

## References

[1] Ramamoorthy C.V., Li H.F. (1977). Pipeline architecture. ACM Comput. Surv. (CSUR).

[2] Gaitan V.G., Gaitan N.C., Ungurean I. (2014). CPU architecture based on a hardware scheduler and independent pipeline registers. IEEE Trans. Very Large Scale Integr. (VLSI) Syst..

[3] Zolfaghari H., Mustafa H., Nurmi J. Run-to-Completion versus Pipelined: The Case of 100 Gbps Packet Parsing. Proceedings of the 2021 IEEE 22nd International Conference on High Performance Switching and Routing (HPSR).

[4] Zhang T., Linguaglossa L., Giaccone P., Iannone L., Roberts J. (2021). Performance benchmarking of state-of-the-art software switches for NFV. Comput. Netw..

[5] Michel O., Bifulco R., Retvari G., Schmid S. (2021). The programmable data plane: Abstractions, architectures, algorithms, and applications. ACM Comput. Surv. (CSUR).

[6] McKeown N., Anderson T., Balakrishnan H., Parulkar G., Peterson L., Rexford J., Shenker S., Turner J. (2008). OpenFlow: Enabling innovation in campus networks. ACM SIGCOMM Comput. Commun. Rev..

[7] Sha M., Guo Z., Song M. (2021). A Review of FPGA’s Application in High-speed Network Processing. J. Netw. New Media.

[8] Ronconi E., Corna N., Costa A., Garzetti F., Lusardi N., Geraci A. (2022). Multi-COBS: A Novel Algorithm for Byte Stuffing at High Throughput. IEEE Access.

[9] Kekely M., Korenek J. Mapping of P4 match-action tables to FPGA. Proceedings of the 2017 27th International Conference on Field Programmable Logic and Applications (FPL).

[10] Bosshart P., Daly D., Gibb G., Izzard M., McKeown N., Rexford J., Schlesinger C., Talayco D., Vahdat A., Varghese G. (2014). P4: Programming Protocol-Independent Packet Processors. SIGCOMM Comput. Commun. Rev..

[11] Jiang W. Scalable ternary content addressable memory implementation using FPGAs. Proceedings of the Architectures for Networking and Communications Systems.

[12] Bosshart P., Gibb G., Kim H.S., Varghese G., McKeown N., Izzard M., Mujica F., Horowitz M. (2013). Forwarding metamorphosis: Fast programmable match-action processing in hardware for SDN. ACM SIGCOMM Comput. Commun. Rev..

[13] Huang X., Guo Z., Song M., Guo Y. (2021). AccelSDP: A reconfigurable accelerator for software data plane based on FPGA SmartNIC. Electronics.

[14] Sha M., Guo Z., Guo Y., Zeng X. (2022). A high-performance and flexible architecture for accelerating SDN on the MPSoC platform. Micromachines.

[15] Kaufmann A., Peter S., Sharma N.K., Anderson T., Krishnamurthy A. High performance packet processing with flexnic. Proceedings of the Twenty-First International Conference on Architectural Support for Programming Languages and Operating Systems.

[16] Kaufmann A., Peter S., Anderson T., Krishnamurthy A. {FlexNIC}: Rethinking Network {DMA}. Proceedings of the 15th Workshop on Hot Topics in Operating Systems (HotOS XV).

[17] Lin J., Patel K., Stephens B.E., Sivaraman A., Akella A. {PANIC}: A {High-Performance} Programmable {NIC} for Multi-tenant Networks. Proceedings of the 14th USENIX Symposium on Operating Systems Design and Implementation (OSDI 20).

[18] Intel Barefoot Tofino. https://www.intel.com/content/www/us/en/products/details/network-io/intelligent-fabric-processors/tofino-2.html/.

[19] Ozdag R. (2012). Intel® Ethernet Switch FM6000 Series-Software Defined Networking. https://people.ucsc.edu/~warner/Bufs/ethernet-switch-fm6000-sdn-paper.pdf.

[20] Wang T., Yang X., Antichi G., Sivaraman A., Panda A. Isolation mechanisms for High-Speed Packet-Processing pipelines. Proceedings of the 19th USENIX Symposium on Networked Systems Design and Implementation (NSDI 22).

[21] Li J., Sun Z., Yan J., Yang X., Jiang Y., Quan W. (2019). DrawerPipe: A reconfigurable pipeline for network processing on FPGA-based SmartNIC. Electronics.

[22] Zolfaghari H., Rossi D., Nurmi J. Reducing crossbar costs in the match-action pipeline. Proceedings of the 2019 IEEE 20th International Conference on High Performance Switching and Routing (HPSR).

[23] Kobiersky P., Korenek J., Polcák L. Packet header analysis and field extraction for multigigabit networks. Proceedings of the 2009 12th International Symposium on Design and Diagnostics of Electronic Circuits & Systems.

[24] Chole S., Fingerhut A., Ma S., Sivaraman A., Vargaftik S., Berger A., Mendelson G., Alizadeh M., Chuang S.T., Keslassy I. drmt: Disaggregated programmable switching. Proceedings of the Conference of the ACM Special Interest Group on Data Communication.

[25] Irfan M., Sanka A.I., Ullah Z., Cheung R.C. (2022). Reconfigurable content-addressable memory (CAM) on FPGAs: A tutorial and survey. Future Gener. Comput. Syst..

[26] Forencich A., Snoeren A.C., Porter G., Papen G. Corundum: An Open-Source 100-Gbps NIC. Proceedings of the 2020 IEEE 28th Annual International Symposium on Field-Programmable Custom Computing Machines (FCCM).

[27] Xilinx (2024). Alveo U200 and U250 Data Center Accelerator Cards Data Sheet (DS962). https://docs.xilinx.com/r/en-US/ds962-u200-u250.

